# Tetra-*O*-4-methyl­phenyl­sulfonyl­penta­erythritol

**DOI:** 10.1107/S1600536808020643

**Published:** 2008-07-12

**Authors:** Shu-Xian Li, Lin Zhu, Hoong-Kun Fun, Suchada Chantrapromma

**Affiliations:** a Department of Chemistry, Handan College, Handan, Hebei 056005, People’s Republic of China; b Department of Chemistry, Beijing Normal University, Beijing 100875, People’s Republic of China; c X-ray Crystallography Unit, School of Physics, Universiti Sains Malaysia, 11800 USM, Penang, Malaysia; d Crystal Materials Research Unit, Department of Chemistry, Faculty of Science, Prince of Songkla University, Hat-Yai, Songkhla 90112, Thailand

## Abstract

In the title mol­ecule (systematic name: methane­tetra­yltetra­methyl­ene tetra-*p*-toluene­sulfonate), C_33_H_36_O_12_S_4_, the central C atom and the S atoms exhibit distorted tetra­hedral configurations. The aromatic rings in opposite arms are nearly parallel to each other, with a dihedral angle of 10.26 (8) or 3.45 (9)°. The mol­ecules are linked into a two-dimensional network parallel to the *bc* plane by weak C—H⋯O hydrogen bonds, π–π [centroid–centroid distance = 3.5806 (12) Å] and S—O⋯π [O⋯centroid = 3.1455 (15) Å and S—O⋯centroid = 122.41 (7)°] inter­molecular inter­actions. Intramolecular C—H⋯O hydrogen bonds are also present.

## Related literature

For bond-length data, see: Allen *et al.* (1987 ▶). For a related structure, see: Li *et al.* (2008 ▶). For general background and applications of penta­erythritol derivatives, see: Constable *et al.* (1998 ▶); Fundueanu *et al.* (1998 ▶); Jiang *et al.* (2002 ▶); Kim *et al.* (2000 ▶); Luo & Chen (2001 ▶); Mischiati *et al.* (2001 ▶); Oike *et al.* (2001 ▶).
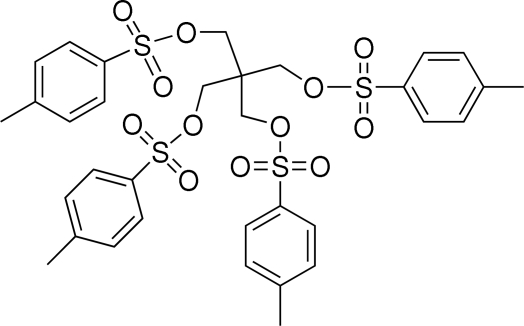


## Experimental

### 

#### Crystal data

C_33_H_36_O_12_S_4_
*M* *_r_* = 752.86
Monoclinic, 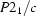

*a* = 13.2983 (2) Å
*b* = 18.0368 (2) Å
*c* = 15.4181 (2) Å
β = 110.653 (1)°
*V* = 3460.50 (8) Å^3^
*Z* = 4
Mo *K*α radiation
μ = 0.34 mm^−1^
*T* = 100.0 (1) K
0.47 × 0.41 × 0.16 mm

#### Data collection

Bruker SMART APEXII CCD area-detector diffractometer
Absorption correction: multi-scan (*SADABS*; Bruker, 2005 ▶) *T*_min_ = 0.857, *T*_max_ = 0.949
45048 measured reflections
10090 independent reflections
7927 reflections with *I* > 2σ(*I*)
*R*_int_ = 0.039

#### Refinement

*R*[*F* ^2^ > 2σ(*F* ^2^)] = 0.043
*wR*(*F* ^2^) = 0.119
*S* = 1.04
10090 reflections
446 parameters
H-atom parameters constrained
Δρ_max_ = 0.66 e Å^−3^
Δρ_min_ = −0.42 e Å^−3^

### 

Data collection: *APEX2* (Bruker, 2005 ▶); cell refinement: *APEX2*; data reduction: *SAINT* (Bruker, 2005 ▶); program(s) used to solve structure: *SHELXTL* (Sheldrick, 2008 ▶); program(s) used to refine structure: *SHELXTL*; molecular graphics: *SHELXTL*; software used to prepare material for publication: *SHELXTL* and *PLATON* (Spek, 2003 ▶).

## Supplementary Material

Crystal structure: contains datablocks global, I. DOI: 10.1107/S1600536808020643/ci2625sup1.cif

Structure factors: contains datablocks I. DOI: 10.1107/S1600536808020643/ci2625Isup2.hkl

Additional supplementary materials:  crystallographic information; 3D view; checkCIF report

## Figures and Tables

**Table 1:** Hydrogen-bond geometry (Å, °)

*D*—H⋯*A*	*D*—H	H⋯*A*	*D*⋯*A*	*D*—H⋯*A*
C2—H2*C*⋯O4	0.97	2.48	2.849 (2)	102
C3—H3*B*⋯O6	0.97	2.47	2.905 (2)	107
C3—H3*B*⋯O7	0.97	2.55	2.876 (2)	100
C3—H3*C*⋯O9^i^	0.97	2.46	3.433 (2)	175
C4—H4*A*⋯O4	0.97	2.55	2.879 (2)	100
C5—H5*B*⋯O12	0.97	2.44	2.888 (2)	107
C5—H5*C*⋯O7	0.97	2.49	2.8396 (19)	101
C7—H7*A*⋯O2	0.93	2.58	2.937 (2)	103
C8—H8*B*⋯O11^ii^	0.93	2.52	3.140 (2)	124
C10—H10*A*⋯O6^i^	0.93	2.41	3.257 (2)	151
C18—H18*A*⋯O6	0.93	2.59	2.932 (2)	103
C22—H22*A*⋯O12^iii^	0.93	2.52	3.154 (2)	126
C25—H25*A*⋯O8	0.93	2.56	2.924 (2)	104
C28—H28*A*⋯O12	0.93	2.54	2.905 (2)	104
C29—H29*A*⋯O8^iv^	0.93	2.42	3.334 (2)	165
C31—H31*A*⋯O12^iii^	0.93	2.53	3.209 (2)	130

Symmetry codes: (i) 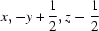; (ii) 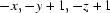; (iii) 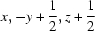; (iv) 
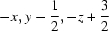
.

**Fig. 1.**
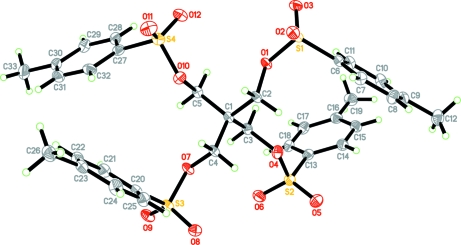
The molecular structure of the title compound, showing 40% probability displacement ellipsoids and the atomic numbering.

**Fig. 2.**
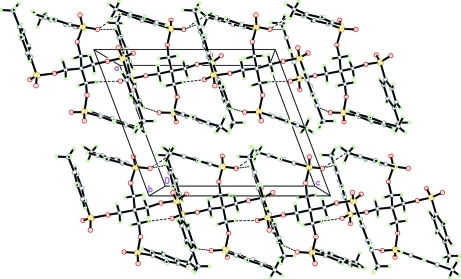
The crystal packing of the title compound, viewed down the b axis. Hydrogen bonds are shown as dashed lines.

## supplementary crystallographic information

### Comment

Pentaerythritol is a valuable starting point for building complex molecular and supramolecular structures due to its symmetric four-armed geometry and is widely used in macromolecular chemistry (Oike *et al.*, 2001), medicinal chemistry (Mischiati *et al.*, 2001; Fundueanu *et al.*, 1998), in the construction of dendrimers (Jiang *et al.*, 2002; Constable *et al.*, 1998) and other applications (Kim *et al.*, 2000; Luo & Chen, 2001). To explore the potential of the pentaerythritol unit in supramolecular chemistry, we report herein the synthesis and crystal structure of tetra-*O*-(4-methylphenylsulfonyl)pentaerythritol, the title compound.

In the title molecule (Fig. 1), atoms C1, S1, S2, S3 and S4 exhibit the usual distorted tetrahedral configuration. The aromatic rings in opposite arms of the molecule are nearly parallel to each other; the dihedral angles between the C6–C11 (A) and  C20–C25 (B) benzene rings is 10.26 (8)° and that between the C13–C18 (C) and C27–C32 (D) benzene rings is 3.45 (9)°. The dihedral angle between the adjacent benzene rings are: A/C 49.67 (9)°, A/D 52.93 (9)°, B/C 53.20 (9)° and B/D 56.15 (9)°. The O1/O7/C1/C2/C4 plane (r.m.s. deviation 0.039 Å) forms dihedral angles of 81.59 (5)° and 84.85 (5)°, respectively, with the rings A and B. The benzene rings C and D form dihedral angles of 59.75 (5)° and 62.82 (5)°, respectively, with the O4/O10/C1/C3/C5 plane. The conformations of the four 4-methylphenylsulfonyl groups with respect to the pentaerythritol unit (C1–C5/O1/O4/O7/O10) can be indicated by torsion angles S1—O1—C2—C1 = -175.72 (10)°, S2—O4—C3—C1 = 154.00 (10)°, S3—O7—C4—C1 = -179.78 (10)° and S4—O10—C5—C1 = 154.75 (10)°. Bond lengths and angles in the title molecule are in normal ranges (Allen *et al.*, 1987) and comparable to those in a related structure (Li *et al.*, 2008).

In the crystal packing (Fig. 2), the molecules are linked into a two-dimensional network parallel to the *bc* plane by weak C—H···O hydrogen bonds (Table 1). In addition, π-π interactions are observed between C13-C18 (centroid Cg1) and C27-C32 (centroid Cg2) benzene rings at (x, y, z) and (1+x, y, z), respectively, with centroid-centroid distance of 3.5806 (12) Å. Also, an S—O···π intermolecular interaction is observed [O3···Cg2^i^ = 3.1455 (15) Å and S1—O3···Cg2^i^ = 122.41 (7)°; the symmetry code is given in Table 1].

### Experimental

The title compound was synthesized by dissolving pentaerythritol (1.36 g, 10.0 mmol) in dry pyridine (80 ml) and tosyl chloride (9.5 g, 50.0 mmol) was then added. The reaction mixture was stirred for 24 h at room temperature, after which it was poured into ice-water (250 ml) containing 1 M HCl and extracted with CH_2_Cl_2_ (80 × 3 ml). The organic layer was washed with water (60 × 2 ml), dried with MgSO_4_ and concentrated. The solid residue was recrystallized from ethanol to afford the desired compound as a white solid (6.42 g, yield: 90%). Block-shaped colourless single crystals of the title compound suitable for *X*-ray structure determination were recrystallized from ethanol by slow evaporation of the solvent in the open air at room temperature (m.p. 423 K).

### Refinement

All H atoms were placed in calculated positions, with C-H = 0.93 Å, *U*_iso_ = 1.2*U*_eq_(C) for aromatic, C-H = 0.97 Å, *U*_iso_ = 1.2*U*_eq_(C) for CH_2_ and C-H = 0.96 Å, *U*_iso_ = 1.5*U*_eq_(C) for CH_3_ atoms. A rotating group model was used for the methyl groups.

### Crystal data

**Table:**

C_33_H_36_O_12_S_4_	*F*_000_ = 1576
*M**_r_* = 752.86	*D*_x_ = 1.445 Mg m^−^^3^
Monoclinic, *P*2_1_/*c*	Melting point: 423 K
Hall symbol: -P 2ybc	Mo *K*α radiation λ = 0.71073 Å
*a* = 13.2983 (2) Å	Cell parameters from 10090 reflections
*b* = 18.0368 (2) Å	θ = 1.8–30.0º
*c* = 15.4181 (2) Å	µ = 0.34 mm^−^^1^
β = 110.653 (1)º	*T* = 100.0 (1) K
*V* = 3460.50 (8) Å^3^	Block, colourless
*Z* = 4	0.47 × 0.41 × 0.16 mm

### Data collection

**Table:**

Bruker SMART APEXII CCD area-detector diffractometer	10090 independent reflections
Radiation source: fine-focus sealed tube	7927 reflections with *I* > 2σ(*I*)
Monochromator: graphite	*R*_int_ = 0.039
Detector resolution: 8.33 pixels mm^-1^	θ_max_ = 30.0º
*T* = 100.0(1) K	θ_min_ = 1.8º
ω scans	*h* = −18→17
Absorption correction: multi-scan(SADABS; Bruker, 2005)	*k* = −25→25
*T*_min_ = 0.857, *T*_max_ = 0.949	*l* = −21→21
45048 measured reflections	

### Refinement

**Table:**

Refinement on *F*^2^	Secondary atom site location: difference Fourier map
Least-squares matrix: full	Hydrogen site location: inferred from neighbouring sites
*R*[*F*^2^ > 2σ(*F*^2^)] = 0.043	H-atom parameters constrained
*wR*(*F*^2^) = 0.119	*w* = 1/[σ^2^(*F*_o_^2^) + (0.0595*P*)^2^ + 1.2746*P*] where *P* = (*F*_o_^2^ + 2*F*_c_^2^)/3
*S* = 1.04	(Δ/σ)_max_ = 0.001
10090 reflections	Δρ_max_ = 0.66 e Å^−^^3^
446 parameters	Δρ_min_ = −0.42 e Å^−^^3^
Primary atom site location: structure-invariant direct methods	Extinction correction: none

### Special details

**Table:**

Experimental. The low-temparture data was collected with the Oxford Cryosystem Cobra low-temperature attachment.
Geometry. All e.s.d.'s (except the e.s.d. in the dihedral angle between two l.s. planes) are estimated using the full covariance matrix. The cell e.s.d.'s are taken into account individually in the estimation of e.s.d.'s in distances, angles and torsion angles; correlations between e.s.d.'s in cell parameters are only used when they are defined by crystal symmetry. An approximate (isotropic) treatment of cell e.s.d.'s is used for estimating e.s.d.'s involving l.s. planes.
Refinement. Refinement of *F*^2^ against ALL reflections. The weighted *R*-factor *wR* and goodness of fit *S* are based on *F*^2^, conventional *R*-factors *R* are based on *F*, with *F* set to zero for negative *F*^2^. The threshold expression of *F*^2^ > σ(*F*^2^) is used only for calculating *R*-factors(gt) *etc*. and is not relevant to the choice of reflections for refinement. *R*-factors based on *F*^2^ are statistically about twice as large as those based on *F*, and *R*- factors based on ALL data will be even larger.

### Fractional atomic coordinates and isotropic or equivalent isotropic displacement parameters (Å^2^)

**Table:**

	*x*	*y*	*z*	*U*_iso_*/*U*_eq_	
S1	0.04280 (3)	0.38393 (2)	0.37155 (3)	0.01965 (9)	
S2	0.41959 (4)	0.31806 (2)	0.71340 (3)	0.02583 (10)	
S3	0.16570 (3)	0.37598 (2)	0.89436 (3)	0.02097 (9)	
S4	−0.18947 (4)	0.29379 (2)	0.54246 (3)	0.02429 (10)	
O1	0.06272 (10)	0.34535 (6)	0.46843 (7)	0.0207 (2)	
O2	−0.01854 (10)	0.45006 (6)	0.36647 (8)	0.0249 (2)	
O3	0.00233 (10)	0.32556 (7)	0.30634 (8)	0.0267 (3)	
O4	0.30102 (10)	0.34530 (6)	0.65781 (9)	0.0256 (3)	
O5	0.48315 (12)	0.38305 (7)	0.72159 (10)	0.0347 (3)	
O6	0.41903 (11)	0.28056 (7)	0.79505 (9)	0.0323 (3)	
O7	0.14708 (10)	0.33953 (6)	0.79639 (7)	0.0213 (2)	
O8	0.24146 (10)	0.43489 (6)	0.90850 (8)	0.0256 (3)	
O9	0.18879 (11)	0.31437 (6)	0.95619 (8)	0.0280 (3)	
O10	−0.07710 (10)	0.33045 (6)	0.60052 (8)	0.0237 (2)	
O11	−0.26269 (11)	0.35436 (7)	0.52419 (10)	0.0334 (3)	
O12	−0.17766 (11)	0.25301 (7)	0.46728 (8)	0.0302 (3)	
C1	0.11235 (13)	0.34044 (8)	0.63322 (10)	0.0189 (3)	
C2	0.08925 (14)	0.39261 (8)	0.55016 (11)	0.0205 (3)	
H2B	0.0296	0.4252	0.5457	0.025*	
H2C	0.1518	0.4227	0.5563	0.025*	
C3	0.21256 (13)	0.29377 (8)	0.64564 (12)	0.0213 (3)	
H3B	0.2282	0.2619	0.6995	0.026*	
H3C	0.2015	0.2629	0.5915	0.026*	
C4	0.12796 (14)	0.38928 (8)	0.71795 (11)	0.0203 (3)	
H4A	0.1888	0.4222	0.7284	0.024*	
H4B	0.0643	0.4191	0.7090	0.024*	
C5	0.01947 (13)	0.28597 (8)	0.61833 (11)	0.0207 (3)	
H5B	0.0121	0.2538	0.5660	0.025*	
H5C	0.0322	0.2555	0.6730	0.025*	
C6	0.17148 (13)	0.40800 (8)	0.37554 (10)	0.0193 (3)	
C7	0.20491 (14)	0.48153 (9)	0.38533 (12)	0.0246 (3)	
H7A	0.1592	0.5190	0.3905	0.030*	
C8	0.30696 (15)	0.49811 (9)	0.38722 (13)	0.0284 (4)	
H8B	0.3296	0.5473	0.3936	0.034*	
C9	0.37690 (14)	0.44311 (10)	0.37979 (12)	0.0265 (3)	
C10	0.34113 (15)	0.36985 (9)	0.36990 (12)	0.0274 (4)	
H10A	0.3868	0.3324	0.3647	0.033*	
C11	0.23934 (15)	0.35158 (9)	0.36764 (12)	0.0249 (3)	
H11B	0.2165	0.3025	0.3610	0.030*	
C12	0.48782 (16)	0.46125 (12)	0.38123 (15)	0.0365 (4)	
H12B	0.5096	0.5085	0.4104	0.055*	
H12C	0.4874	0.4630	0.3189	0.055*	
H12D	0.5372	0.4238	0.4154	0.055*	
C13	0.44820 (14)	0.25381 (9)	0.64082 (12)	0.0250 (3)	
C14	0.48460 (15)	0.27930 (10)	0.57213 (12)	0.0279 (4)	
H14A	0.4921	0.3298	0.5641	0.033*	
C15	0.50952 (15)	0.22807 (10)	0.51580 (13)	0.0296 (4)	
H15A	0.5344	0.2447	0.4699	0.035*	
C16	0.49800 (15)	0.15206 (10)	0.52657 (13)	0.0290 (4)	
C17	0.46100 (15)	0.12852 (10)	0.59613 (14)	0.0306 (4)	
H17A	0.4530	0.0780	0.6042	0.037*	
C18	0.43601 (14)	0.17829 (9)	0.65301 (13)	0.0266 (3)	
H18A	0.4113	0.1617	0.6990	0.032*	
C19	0.52492 (17)	0.09697 (12)	0.46446 (14)	0.0370 (4)	
H19A	0.5668	0.0573	0.5013	0.056*	
H19B	0.5654	0.1212	0.4320	0.056*	
H19C	0.4597	0.0773	0.4205	0.056*	
C20	0.04014 (14)	0.41323 (8)	0.88336 (11)	0.0211 (3)	
C21	−0.04285 (15)	0.36589 (9)	0.88235 (12)	0.0262 (4)	
H21A	−0.0325	0.3148	0.8863	0.031*	
C22	−0.14120 (15)	0.39600 (10)	0.87544 (12)	0.0275 (4)	
H22A	−0.1969	0.3646	0.8750	0.033*	
C23	−0.15853 (15)	0.47227 (10)	0.86912 (12)	0.0257 (3)	
C24	−0.07485 (16)	0.51828 (10)	0.86920 (13)	0.0295 (4)	
H24A	−0.0855	0.5693	0.8647	0.035*	
C25	0.02422 (15)	0.48965 (9)	0.87594 (12)	0.0267 (4)	
H25A	0.0795	0.5211	0.8755	0.032*	
C26	−0.26526 (16)	0.50341 (11)	0.86390 (14)	0.0345 (4)	
H26A	−0.2727	0.5529	0.8395	0.052*	
H26B	−0.2694	0.5043	0.9248	0.052*	
H26C	−0.3219	0.4729	0.8240	0.052*	
C27	−0.21526 (13)	0.23237 (9)	0.61915 (12)	0.0229 (3)	
C28	−0.21199 (14)	0.15680 (9)	0.60537 (12)	0.0250 (3)	
H28A	−0.1927	0.1383	0.5571	0.030*	
C29	−0.23782 (15)	0.10914 (10)	0.66464 (13)	0.0289 (4)	
H29A	−0.2360	0.0582	0.6556	0.035*	
C30	−0.26628 (15)	0.13558 (11)	0.73704 (13)	0.0294 (4)	
C31	−0.26943 (15)	0.21215 (11)	0.74919 (13)	0.0304 (4)	
H31A	−0.2891	0.2307	0.7972	0.037*	
C32	−0.24395 (15)	0.26053 (10)	0.69117 (12)	0.0278 (4)	
H32A	−0.2459	0.3114	0.7000	0.033*	
C33	−0.29291 (18)	0.08257 (13)	0.80129 (15)	0.0412 (5)	
H33A	−0.3346	0.0421	0.7661	0.062*	
H33B	−0.3335	0.1080	0.8328	0.062*	
H33C	−0.2276	0.0637	0.8460	0.062*	

### Atomic displacement parameters (Å^2^)

**Table:**

	*U*^11^	*U*^22^	*U*^33^	*U*^12^	*U*^13^	*U*^23^
S1	0.0232 (2)	0.02041 (17)	0.01541 (17)	0.00089 (14)	0.00691 (15)	−0.00004 (13)
S2	0.0271 (2)	0.0254 (2)	0.0251 (2)	−0.00384 (16)	0.00944 (17)	−0.00123 (16)
S3	0.0284 (2)	0.01908 (17)	0.01522 (18)	0.00331 (14)	0.00744 (16)	0.00123 (13)
S4	0.0255 (2)	0.02166 (18)	0.0239 (2)	0.00318 (15)	0.00649 (17)	−0.00113 (15)
O1	0.0300 (6)	0.0187 (5)	0.0152 (5)	−0.0004 (4)	0.0103 (5)	−0.0008 (4)
O2	0.0259 (6)	0.0246 (5)	0.0243 (6)	0.0052 (5)	0.0090 (5)	0.0024 (5)
O3	0.0298 (7)	0.0288 (6)	0.0193 (6)	−0.0038 (5)	0.0060 (5)	−0.0053 (5)
O4	0.0273 (6)	0.0218 (5)	0.0310 (6)	0.0007 (5)	0.0143 (5)	0.0025 (5)
O5	0.0381 (8)	0.0302 (6)	0.0361 (7)	−0.0115 (5)	0.0133 (6)	−0.0044 (5)
O6	0.0386 (8)	0.0343 (7)	0.0228 (6)	−0.0049 (6)	0.0093 (6)	0.0004 (5)
O7	0.0321 (7)	0.0177 (5)	0.0151 (5)	0.0052 (4)	0.0097 (5)	0.0022 (4)
O8	0.0265 (6)	0.0254 (6)	0.0227 (6)	0.0006 (5)	0.0057 (5)	−0.0001 (5)
O9	0.0401 (8)	0.0243 (6)	0.0180 (6)	0.0057 (5)	0.0085 (5)	0.0053 (4)
O10	0.0224 (6)	0.0204 (5)	0.0289 (6)	0.0013 (4)	0.0099 (5)	−0.0037 (5)
O11	0.0310 (7)	0.0252 (6)	0.0390 (8)	0.0076 (5)	0.0063 (6)	0.0022 (5)
O12	0.0381 (8)	0.0297 (6)	0.0213 (6)	0.0014 (5)	0.0088 (5)	−0.0021 (5)
C1	0.0248 (8)	0.0175 (6)	0.0161 (7)	0.0020 (6)	0.0091 (6)	0.0007 (5)
C2	0.0287 (9)	0.0184 (6)	0.0165 (7)	0.0010 (6)	0.0107 (6)	−0.0016 (5)
C3	0.0241 (8)	0.0197 (7)	0.0225 (8)	0.0012 (6)	0.0110 (7)	−0.0009 (6)
C4	0.0294 (9)	0.0172 (6)	0.0158 (7)	0.0033 (6)	0.0099 (6)	0.0020 (5)
C5	0.0245 (8)	0.0182 (6)	0.0212 (7)	0.0027 (6)	0.0103 (7)	0.0015 (6)
C6	0.0240 (8)	0.0199 (7)	0.0154 (7)	0.0012 (6)	0.0088 (6)	0.0012 (5)
C7	0.0283 (9)	0.0188 (7)	0.0278 (8)	0.0016 (6)	0.0112 (7)	−0.0021 (6)
C8	0.0286 (9)	0.0227 (7)	0.0356 (10)	−0.0018 (6)	0.0133 (8)	−0.0006 (7)
C9	0.0257 (9)	0.0316 (8)	0.0244 (8)	0.0025 (7)	0.0115 (7)	0.0035 (7)
C10	0.0327 (10)	0.0254 (8)	0.0293 (9)	0.0092 (7)	0.0175 (8)	0.0048 (7)
C11	0.0348 (10)	0.0178 (7)	0.0263 (8)	0.0033 (6)	0.0159 (7)	0.0018 (6)
C12	0.0290 (10)	0.0412 (10)	0.0443 (12)	0.0038 (8)	0.0190 (9)	0.0072 (9)
C13	0.0197 (8)	0.0280 (8)	0.0260 (8)	0.0004 (6)	0.0066 (7)	−0.0001 (7)
C14	0.0246 (9)	0.0318 (8)	0.0254 (8)	0.0006 (7)	0.0066 (7)	0.0050 (7)
C15	0.0270 (9)	0.0374 (9)	0.0237 (8)	0.0020 (7)	0.0083 (7)	0.0040 (7)
C16	0.0222 (9)	0.0348 (9)	0.0281 (9)	0.0030 (7)	0.0064 (7)	−0.0026 (7)
C17	0.0265 (9)	0.0282 (8)	0.0381 (10)	0.0001 (7)	0.0125 (8)	−0.0001 (7)
C18	0.0221 (8)	0.0274 (8)	0.0317 (9)	−0.0013 (6)	0.0111 (7)	0.0012 (7)
C19	0.0365 (11)	0.0414 (10)	0.0337 (10)	0.0048 (8)	0.0130 (9)	−0.0035 (8)
C20	0.0285 (9)	0.0197 (7)	0.0169 (7)	0.0023 (6)	0.0105 (6)	0.0002 (6)
C21	0.0354 (10)	0.0204 (7)	0.0254 (8)	−0.0007 (6)	0.0140 (7)	0.0004 (6)
C22	0.0319 (10)	0.0280 (8)	0.0252 (8)	−0.0043 (7)	0.0130 (7)	−0.0007 (7)
C23	0.0311 (9)	0.0290 (8)	0.0191 (8)	0.0028 (7)	0.0116 (7)	−0.0006 (6)
C24	0.0381 (10)	0.0216 (7)	0.0336 (9)	0.0063 (7)	0.0185 (8)	0.0012 (7)
C25	0.0331 (10)	0.0205 (7)	0.0295 (9)	−0.0013 (6)	0.0149 (8)	0.0004 (6)
C26	0.0344 (11)	0.0411 (10)	0.0322 (10)	0.0068 (8)	0.0168 (8)	−0.0009 (8)
C27	0.0178 (8)	0.0248 (7)	0.0242 (8)	0.0014 (6)	0.0051 (6)	−0.0021 (6)
C28	0.0228 (8)	0.0244 (8)	0.0300 (9)	0.0010 (6)	0.0120 (7)	−0.0043 (6)
C29	0.0254 (9)	0.0257 (8)	0.0373 (10)	−0.0005 (6)	0.0133 (8)	−0.0007 (7)
C30	0.0204 (9)	0.0401 (10)	0.0284 (9)	−0.0018 (7)	0.0093 (7)	0.0017 (7)
C31	0.0254 (9)	0.0430 (10)	0.0236 (8)	0.0032 (7)	0.0097 (7)	−0.0056 (7)
C32	0.0272 (9)	0.0296 (8)	0.0238 (8)	0.0034 (7)	0.0056 (7)	−0.0064 (7)
C33	0.0358 (11)	0.0524 (12)	0.0406 (12)	−0.0050 (9)	0.0198 (9)	0.0083 (10)

### Geometric parameters (Å, °)

**Table:**

S1—O3	1.4238 (12)	C12—H12C	0.96
S1—O2	1.4315 (12)	C12—H12D	0.96
S1—O1	1.5833 (11)	C13—C14	1.389 (2)
S1—C6	1.7453 (17)	C13—C18	1.392 (2)
S2—O5	1.4244 (13)	C14—C15	1.386 (3)
S2—O6	1.4314 (13)	C14—H14A	0.93
S2—O4	1.5840 (13)	C15—C16	1.396 (3)
S2—C13	1.7435 (18)	C15—H15A	0.93
S3—O9	1.4252 (12)	C16—C17	1.395 (3)
S3—O8	1.4271 (13)	C16—C19	1.508 (3)
S3—O7	1.5848 (11)	C17—C18	1.375 (3)
S3—C20	1.7518 (17)	C17—H17A	0.93
S4—O11	1.4240 (13)	C18—H18A	0.93
S4—O12	1.4274 (13)	C19—H19A	0.96
S4—O10	1.5899 (13)	C19—H19B	0.96
S4—C27	1.7404 (18)	C19—H19C	0.96
O1—C2	1.4578 (18)	C20—C21	1.391 (2)
O4—C3	1.458 (2)	C20—C25	1.393 (2)
O7—C4	1.4546 (18)	C21—C22	1.385 (3)
O10—C5	1.4559 (19)	C21—H21A	0.93
C1—C4	1.528 (2)	C22—C23	1.393 (2)
C1—C3	1.530 (2)	C22—H22A	0.93
C1—C2	1.531 (2)	C23—C24	1.388 (3)
C1—C5	1.531 (2)	C23—C26	1.502 (3)
C2—H2B	0.97	C24—C25	1.384 (3)
C2—H2C	0.97	C24—H24A	0.93
C3—H3B	0.97	C25—H25A	0.93
C3—H3C	0.97	C26—H26A	0.96
C4—H4A	0.97	C26—H26B	0.96
C4—H4B	0.97	C26—H26C	0.96
C5—H5B	0.97	C27—C28	1.383 (2)
C5—H5C	0.97	C27—C32	1.391 (2)
C6—C7	1.390 (2)	C28—C29	1.383 (2)
C6—C11	1.394 (2)	C28—H28A	0.93
C7—C8	1.380 (2)	C29—C30	1.384 (3)
C7—H7A	0.93	C29—H29A	0.93
C8—C9	1.392 (2)	C30—C31	1.396 (3)
C8—H8B	0.93	C30—C33	1.506 (3)
C9—C10	1.395 (2)	C31—C32	1.375 (3)
C9—C12	1.503 (3)	C31—H31A	0.93
C10—C11	1.382 (3)	C32—H32A	0.93
C10—H10A	0.93	C33—H33A	0.96
C11—H11B	0.93	C33—H33B	0.96
C12—H12B	0.96	C33—H33C	0.96
			
O3—S1—O2	120.75 (8)	C9—C12—H12C	109.5
O3—S1—O1	103.71 (7)	H12B—C12—H12C	109.5
O2—S1—O1	108.68 (7)	C9—C12—H12D	109.5
O3—S1—C6	109.30 (8)	H12B—C12—H12D	109.5
O2—S1—C6	109.11 (7)	H12C—C12—H12D	109.5
O1—S1—C6	103.91 (7)	C14—C13—C18	121.01 (16)
O5—S2—O6	119.85 (8)	C14—C13—S2	118.89 (13)
O5—S2—O4	103.83 (8)	C18—C13—S2	120.09 (14)
O6—S2—O4	108.03 (8)	C15—C14—C13	118.81 (17)
O5—S2—C13	110.30 (9)	C15—C14—H14A	120.6
O6—S2—C13	108.69 (8)	C13—C14—H14A	120.6
O4—S2—C13	105.06 (8)	C14—C15—C16	121.31 (17)
O9—S3—O8	120.30 (8)	C14—C15—H15A	119.3
O9—S3—O7	103.79 (7)	C16—C15—H15A	119.3
O8—S3—O7	108.60 (7)	C17—C16—C15	118.28 (17)
O9—S3—C20	109.69 (8)	C17—C16—C19	120.99 (17)
O8—S3—C20	109.06 (7)	C15—C16—C19	120.73 (17)
O7—S3—C20	104.11 (7)	C18—C17—C16	121.45 (17)
O11—S4—O12	119.87 (8)	C18—C17—H17A	119.3
O11—S4—O10	103.51 (7)	C16—C17—H17A	119.3
O12—S4—O10	108.71 (7)	C17—C18—C13	119.13 (17)
O11—S4—C27	109.74 (8)	C17—C18—H18A	120.4
O12—S4—C27	109.16 (8)	C13—C18—H18A	120.4
O10—S4—C27	104.68 (7)	C16—C19—H19A	109.5
C2—O1—S1	117.87 (9)	C16—C19—H19B	109.5
C3—O4—S2	118.41 (10)	H19A—C19—H19B	109.5
C4—O7—S3	117.34 (9)	C16—C19—H19C	109.5
C5—O10—S4	117.92 (10)	H19A—C19—H19C	109.5
C4—C1—C3	111.13 (13)	H19B—C19—H19C	109.5
C4—C1—C2	106.71 (12)	C21—C20—C25	120.63 (16)
C3—C1—C2	110.60 (13)	C21—C20—S3	119.40 (12)
C4—C1—C5	110.94 (13)	C25—C20—S3	119.97 (13)
C3—C1—C5	106.66 (12)	C22—C21—C20	118.94 (15)
C2—C1—C5	110.85 (13)	C22—C21—H21A	120.5
O1—C2—C1	106.24 (11)	C20—C21—H21A	120.5
O1—C2—H2B	110.5	C21—C22—C23	121.42 (17)
C1—C2—H2B	110.5	C21—C22—H22A	119.3
O1—C2—H2C	110.5	C23—C22—H22A	119.3
C1—C2—H2C	110.5	C24—C23—C22	118.54 (17)
H2B—C2—H2C	108.7	C24—C23—C26	121.25 (16)
O4—C3—C1	107.03 (12)	C22—C23—C26	120.21 (17)
O4—C3—H3B	110.3	C25—C24—C23	121.23 (16)
C1—C3—H3B	110.3	C25—C24—H24A	119.4
O4—C3—H3C	110.3	C23—C24—H24A	119.4
C1—C3—H3C	110.3	C24—C25—C20	119.23 (17)
H3B—C3—H3C	108.6	C24—C25—H25A	120.4
O7—C4—C1	106.63 (11)	C20—C25—H25A	120.4
O7—C4—H4A	110.4	C23—C26—H26A	109.5
C1—C4—H4A	110.4	C23—C26—H26B	109.5
O7—C4—H4B	110.4	H26A—C26—H26B	109.5
C1—C4—H4B	110.4	C23—C26—H26C	109.5
H4A—C4—H4B	108.6	H26A—C26—H26C	109.5
O10—C5—C1	106.62 (12)	H26B—C26—H26C	109.5
O10—C5—H5B	110.4	C28—C27—C32	120.99 (16)
C1—C5—H5B	110.4	C28—C27—S4	119.91 (13)
O10—C5—H5C	110.4	C32—C27—S4	119.03 (13)
C1—C5—H5C	110.4	C27—C28—C29	118.85 (16)
H5B—C5—H5C	108.6	C27—C28—H28A	120.6
C7—C6—C11	120.93 (16)	C29—C28—H28A	120.6
C7—C6—S1	120.88 (13)	C28—C29—C30	121.41 (16)
C11—C6—S1	118.18 (12)	C28—C29—H29A	119.3
C8—C7—C6	118.92 (15)	C30—C29—H29A	119.3
C8—C7—H7A	120.5	C29—C30—C31	118.61 (17)
C6—C7—H7A	120.5	C29—C30—C33	120.43 (18)
C7—C8—C9	121.64 (16)	C31—C30—C33	120.96 (18)
C7—C8—H8B	119.2	C32—C31—C30	120.96 (17)
C9—C8—H8B	119.2	C32—C31—H31A	119.5
C8—C9—C10	118.19 (16)	C30—C31—H31A	119.5
C8—C9—C12	121.62 (17)	C31—C32—C27	119.18 (16)
C10—C9—C12	120.19 (16)	C31—C32—H32A	120.4
C11—C10—C9	121.47 (16)	C27—C32—H32A	120.4
C11—C10—H10A	119.3	C30—C33—H33A	109.5
C9—C10—H10A	119.3	C30—C33—H33B	109.5
C10—C11—C6	118.85 (15)	H33A—C33—H33B	109.5
C10—C11—H11B	120.6	C30—C33—H33C	109.5
C6—C11—H11B	120.6	H33A—C33—H33C	109.5
C9—C12—H12B	109.5	H33B—C33—H33C	109.5
			
O3—S1—O1—C2	−170.85 (11)	O4—S2—C13—C14	83.71 (15)
O2—S1—O1—C2	−41.20 (13)	O5—S2—C13—C18	151.46 (14)
C6—S1—O1—C2	74.89 (12)	O6—S2—C13—C18	18.19 (17)
O5—S2—O4—C3	−173.46 (11)	O4—S2—C13—C18	−97.23 (15)
O6—S2—O4—C3	−45.20 (13)	C18—C13—C14—C15	−0.4 (3)
C13—S2—O4—C3	70.68 (13)	S2—C13—C14—C15	178.62 (14)
O9—S3—O7—C4	−176.31 (12)	C13—C14—C15—C16	0.4 (3)
O8—S3—O7—C4	−47.20 (13)	C14—C15—C16—C17	−0.3 (3)
C20—S3—O7—C4	68.89 (13)	C14—C15—C16—C19	179.71 (18)
O11—S4—O10—C5	−170.33 (11)	C15—C16—C17—C18	0.1 (3)
O12—S4—O10—C5	−41.85 (13)	C19—C16—C17—C18	−179.86 (18)
C27—S4—O10—C5	74.70 (12)	C16—C17—C18—C13	−0.1 (3)
S1—O1—C2—C1	−175.72 (10)	C14—C13—C18—C17	0.3 (3)
C4—C1—C2—O1	−173.83 (13)	S2—C13—C18—C17	−178.76 (14)
C3—C1—C2—O1	65.17 (16)	O9—S3—C20—C21	−37.17 (15)
C5—C1—C2—O1	−52.92 (16)	O8—S3—C20—C21	−170.84 (13)
S2—O4—C3—C1	154.00 (10)	O7—S3—C20—C21	73.38 (14)
C4—C1—C3—O4	−60.20 (16)	O9—S3—C20—C25	142.69 (14)
C2—C1—C3—O4	58.14 (16)	O8—S3—C20—C25	9.02 (16)
C5—C1—C3—O4	178.76 (12)	O7—S3—C20—C25	−106.76 (14)
S3—O7—C4—C1	−179.78 (10)	C25—C20—C21—C22	−1.1 (3)
C3—C1—C4—O7	−60.81 (16)	S3—C20—C21—C22	178.76 (13)
C2—C1—C4—O7	178.53 (13)	C20—C21—C22—C23	0.3 (3)
C5—C1—C4—O7	57.68 (16)	C21—C22—C23—C24	0.5 (3)
S4—O10—C5—C1	154.75 (10)	C21—C22—C23—C26	−178.59 (17)
C4—C1—C5—O10	62.45 (15)	C22—C23—C24—C25	−0.4 (3)
C3—C1—C5—O10	−176.39 (12)	C26—C23—C24—C25	178.66 (16)
C2—C1—C5—O10	−55.93 (15)	C23—C24—C25—C20	−0.4 (3)
O3—S1—C6—C7	142.11 (13)	C21—C20—C25—C24	1.2 (3)
O2—S1—C6—C7	8.11 (16)	S3—C20—C25—C24	−178.69 (13)
O1—S1—C6—C7	−107.68 (14)	O11—S4—C27—C28	138.81 (14)
O3—S1—C6—C11	−37.20 (15)	O12—S4—C27—C28	5.58 (17)
O2—S1—C6—C11	−171.20 (12)	O10—S4—C27—C28	−110.67 (14)
O1—S1—C6—C11	73.01 (14)	O11—S4—C27—C32	−38.17 (16)
C11—C6—C7—C8	−0.1 (2)	O12—S4—C27—C32	−171.40 (14)
S1—C6—C7—C8	−179.35 (13)	O10—S4—C27—C32	72.35 (15)
C6—C7—C8—C9	−0.1 (3)	C32—C27—C28—C29	0.0 (3)
C7—C8—C9—C10	0.2 (3)	S4—C27—C28—C29	−176.92 (14)
C7—C8—C9—C12	179.59 (17)	C27—C28—C29—C30	−0.2 (3)
C8—C9—C10—C11	−0.1 (3)	C28—C29—C30—C31	0.5 (3)
C12—C9—C10—C11	−179.50 (17)	C28—C29—C30—C33	−179.33 (18)
C9—C10—C11—C6	0.0 (3)	C29—C30—C31—C32	−0.6 (3)
C7—C6—C11—C10	0.2 (2)	C33—C30—C31—C32	179.26 (18)
S1—C6—C11—C10	179.46 (13)	C30—C31—C32—C27	0.4 (3)
O5—S2—C13—C14	−27.60 (17)	C28—C27—C32—C31	−0.1 (3)
O6—S2—C13—C14	−160.86 (14)	S4—C27—C32—C31	176.88 (14)

### Hydrogen-bond geometry (Å, °)

**Table:**

*D*—H···*A*	*D*—H	H···*A*	*D*···*A*	*D*—H···*A*
C2—H2C···O4	0.97	2.48	2.849 (2)	102
C3—H3B···O6	0.97	2.47	2.905 (2)	107
C3—H3B···O7	0.97	2.55	2.876 (2)	100
C3—H3C···O9^i^	0.97	2.46	3.433 (2)	175
C4—H4A···O4	0.97	2.55	2.879 (2)	100
C5—H5B···O12	0.97	2.44	2.888 (2)	107
C5—H5C···O7	0.97	2.49	2.8396 (19)	101
C7—H7A···O2	0.93	2.58	2.937 (2)	103
C8—H8B···O11^ii^	0.93	2.52	3.140 (2)	124
C10—H10A···O6^i^	0.93	2.41	3.257 (2)	151
C18—H18A···O6	0.93	2.59	2.932 (2)	103
C22—H22A···O12^iii^	0.93	2.52	3.154 (2)	126
C25—H25A···O8	0.93	2.56	2.924 (2)	104
C28—H28A···O12	0.93	2.54	2.905 (2)	104
C29—H29A···O8^iv^	0.93	2.42	3.334 (2)	165
C31—H31A···O12^iii^	0.93	2.53	3.209 (2)	130

Symmetry codes:  (i) *x*, −*y*+1/2, *z*−1/2; (ii) −*x*, −*y*+1, −*z*+1; (iii) *x*, −*y*+1/2, *z*+1/2; (iv) −*x*, *y*−1/2, −*z*+3/2.
